# Advanced Nanomaterials and Nanotechnology: Highlights from the 2025 Editor’s Choice Collection

**DOI:** 10.3390/ma19112392

**Published:** 2026-06-04

**Authors:** Carlos Lodeiro

**Affiliations:** 1 BIOSCOPE Research Group, OMICS & Analytical Development Group, Associated Laboratory for Green Chemistry LAQV-REQUIMTE, NOVA FCT, Universidade NOVA de Lisboa, 2829-516 Caparica, Portugal; cle@fct.unl.pt or clodeiro@bioscopegroup.org; 2 PROTEOMASS Scientific Society, 2829-466 Costa de Caparica, Portugal

Nanotechnology continues to redefine the boundaries of science, medicine, energy, and environmental stewardship. By engineering matter below 100 nm, researchers unlock quantum, optical, electronic, and catalytic properties that bulk materials cannot replicate [1,2]. The deliberate manipulation of composition, morphology, and surface chemistry at the nanoscale gives rise to phenomena that have no counterpart in macroscopic solids—plasmon resonance, quantum confinement, superparamagnetism, and anomalous catalytic reactivity [3,4]. These distinctive features have propelled nanomaterials to the forefront of several of the most pressing technological challenges of the 21st century, from sustainable energy generation and conversion [5,6] to precision medicine [7], environmental remediation [8,9], and smart sensing, gas-monitoring, and soft robotic systems [10,11,12].

In the energy sector, nanotechnology is transforming both conversion and storage. Transition-metal layered double hydroxide nanosheets, micro/nanofluidic interfaces, and engineered nanoscale architectures are driving improvements in water splitting, biomass upgrading, aqueous energy conversion, and related green energy processes [5,6,13,14]. Nanostructured semiconductors and advanced carbon-based systems contribute to energy-efficient devices, high-surface-area electrodes, and improved charge-transfer pathways in batteries and electrochemical systems [15,16,17,18,19]. Meanwhile, the emergence of organic and bipolar molecular electrode materials offers a sustainability-oriented alternative to cobalt- and nickel-dependent inorganic cathodes, as highlighted by one of the selected contributions discussed below through combined density functional theory and electrochemical impedance spectroscopy.

In medicine and biology, nanomaterials are enabling diagnostic and therapeutic capabilities that conventional molecular agents cannot match. Surface-engineered lanthanide nanoparticles illustrate how nanoscale composition and ligand chemistry can combine image guidance, phototherapy, radiotherapy, chemotherapy, gene therapy, and immunotherapy in a single oncological platform [7]. Among the selected papers discussed below, radiolabeled nanoparticles combining diagnostic imaging and locoregional radiotherapy exemplify the theranostic paradigm, while multifunctional nanoflakes integrating photosensitizers, radiosensitizers, and MRI contrast agents are advancing multimodal cancer therapy. Biopolymer–graphene nanocomposites spanning chitosan, hyaluronic acid, cellulose, and silk fibroin matrices are finding applications in targeted drug delivery, tissue engineering, wound healing, and antimicrobial systems. The growing ubiquity of engineered nanomaterials has, however, necessitated rigorous nanotoxicological assessment, and one of the highlighted studies shows that co-exposure to silver nanoparticles and nanoplastics produces complex synergistic and antagonistic interactions on viability and gene expression that single-agent studies systematically miss.

Nanomaterials are equally transforming food science, environmental chemistry, and heritage conservation. Nanoarchitectonic food-packaging systems combining polysaccharide nanocomposites, metal nanoparticle-loaded biopolymers, silver/poly(methyl methacrylate) films, and active nano-coatings can extend shelf-life, preserve nutritional quality, tune wettability, and reduce plastic waste across diverse food categories [12]. Multifunctional MOF–nanocellulose composites and defective black TiO_2_ catalysts now define important routes for dye degradation, pharmaceutical removal, volatile organic compound capture, CO_2_ separation, and photocatalytic water remediation [8,9]. The selected papers discussed below further illustrate how electrochemical nitrate reduction can pair water pollution remediation with sustainable ammonia synthesis, how life cycle assessment can become a benchmark for responsible nanomaterial development, and how magnesium hydroxide nanoparticles combined with biopolymer consolidants can strengthen and deacidify fragile archival paper.

Colloidal stability and surface chemistry remain foundational challenges across all application domains. The synthesis and stabilization of anisotropic plasmonic metal nanoparticles—from branched gold nanoparticles with polyallylamine-controlled morphology and tunable localized surface plasmon resonance (LSPR) across the visible-to-near-infrared region [20] to silver nanoplates with precisely engineered dimensions governed by pH, EDTA complexation, and adenosine monophosphate-mediated facet control [21]—underpins applications spanning biosensing, photothermal therapy, and refractive-index probing. Recently, anisotropic gold nanostructures were extended into printable diagnostics using inkjet-printed gold seeds and AMP-directed, seed-mediated growth of gold nanostars on cellulose paper to create a biodegradable, smartphone-readable glucose sensor with a 1–200 µM linear range and a 0.56 µM detection limit [22]. In the antimicrobial domain, silver/poly(methyl methacrylate) films and the systematic incorporation of silver, copper, and bimetallic AgCu nanoparticles into polymeric matrices—via sputtering, solvent casting, electrospinning, spin coating, and plasma polymerization—yield composite materials with tunable wettability, controlled ion release, and multifaceted antimicrobial mechanisms [12,23]. Different international forums have collectively cataloged the rapid evolution of two-dimensional boron nanomaterials [4], hybrid energy-storage devices [24], sustainable energy nanomaterials [25], and smart supercapacitors based on MOF/graphene-oxide derivatives [26] that further underpin both fundamental science and industrial translation.

The “Advanced Nanomaterials and Nanotechnology” Section of *Materials* serves as a dedicated forum for rapid, rigorous publication of original and review articles encompassing the preparation, characterization, and application of all classes of nanomaterials—from nanoparticles and nanocatalysts to nanoporous frameworks, thin films, nanocomposites, and nanoscale devices. This Editorial highlights fourteen influential Editor’s Choice articles published in the Section in 2025. For clarity, the selected papers are ordered below as papers 1–14. Spanning transparent nanoelectronics, nanobrachytherapy, sustainable food packaging, fuel cell nanocatalysts, organic electrode materials, electrochemical nitrate reduction, nanotoxicology, green surfactant design, graphene bio-nanocomposites, lithium–oxygen batteries, solar energy nanostructures, multimodal cancer nanotherapy, plasmonic nanoparticle stabilization, and cultural heritage conservation, these articles collectively illustrate the extraordinary breadth and societal impact of contemporary nanomaterials research. We summarize each contribution, identify cross-cutting themes, and reflect on the directions shaping this dynamic section.

## 1. Transparent Nanoelectronics: High-Mobility Amorphous Oxide Thin-Film Transistors [27]

The demand for high-performance, transparent electronics—spanning flexible displays, augmented-reality optics, and transparent sensors—depends critically on oxide semiconductors that are simultaneously conductive, optically clear, and mechanically robust. Kim et al. [27] report the fabrication of all-transparent thin-film transistors (AT-TFTs) in which a single amorphous indium–zinc–tin-oxide (a-IZTO) film is engineered to serve as both the high-mobility channel layer and the transparent conductive electrode (TCE). By tuning the In/Zn/Sn stoichiometry and exploiting radio-frequency magnetron sputtering, the authors achieve a dual-functional material that eliminates the need for separate electrode and active-layer materials. X-ray diffraction confirms the amorphous character of the films after post-anneal, ensuring uniform carrier transport pathways. The resulting AT-TFTs deliver high field-effect mobility alongside excellent bias-illumination stability, addressing a major barrier to industrial adoption of transparent electronics.

This work is significant because it demonstrates that compositional engineering of a single amorphous oxide can simultaneously optimize electrical mobility and optical transparency—a design principle with broad implications for next-generation displays, solar cells, and wearable devices.

## 2. Nanoscale Brachytherapy: ^161^Terbium-Labeled Gold Nanoparticles Against Breast Cancer [28]

Radionuclide theranostics—where a single agent provides both therapeutic radiation and diagnostic imaging—represent a transformative paradigm in oncology. Salvanou et al. [28] propose an innovative injectable nanobrachytherapy platform for breast cancer, combining gold nanoparticles functionalized with the TADOTAGA chelator (Au@TADOTAGA) and iron oxide nanoflowers (NFAu@TADOTAGA) labeled with terbium-161 (^161^Tb). ^161^Tb emits Auger and conversion electrons alongside beta particles and gamma photons, making it ideally suited for locoregional tumor irradiation and simultaneous imaging. The radiolabeling efficiency was high, and the hybrid nanosystems demonstrated excellent in vitro stability over 21 days post-labeling.

The dual nature of the nanoflower scaffold—providing both radiolabeling sites and magnetic resonance imaging contrast—exemplifies the “multimodal” design philosophy that is rapidly becoming the standard in cancer nanomedicine. The work offers a compelling proof of concept for injectable nanobrachytherapy as an alternative to implanted radioactive seeds.

## 3. Nanoarchitectonics for Sustainable Food Packaging [29]

Food security and plastic pollution are among the defining global challenges of the 21st century. Yang and Skirtach [29] provide the first dedicated review of nanoarchitectonics as applied to sustainable food packaging—the deliberate, hierarchical engineering of nanoscale building blocks to achieve specific macroscopic protection and sensing functions. Their analysis covers the structural and functional hierarchy needed for diverse food categories, from meat and dairy to fruits, vegetables, and beverages, and evaluates how nanoarchitectonics can extend shelf-life, maintain nutritional quality, and reduce plastic waste. Materials considered include polysaccharide nanocomposites, metal nanoparticle-loaded biopolymers, clay nanoplatelets, and active nano-coatings with controlled release of antimicrobials or oxygen scavengers.

The novelty of this work lies in framing food packaging explicitly through the lens of nanoarchitectonics—a concept developed primarily in materials science and nanotechnology—revealing new design principles that purely application-oriented food science has not yet exploited. The authors’ emphasis on biodegradability and regulatory compatibility makes this review immediately actionable for industry.

## 4. One-Pot Pd@Pt Core–Shell Nanocatalysts for Fuel Cell Oxygen Reduction [30]

Proton exchange membrane fuel cells require cathode catalysts that efficiently drive the oxygen reduction reaction (ORR) at minimal platinum loading. Tang et al. [30] report a facile one-pot synthesis of Pd@Pt core–shell icosahedra using triethylene glycol as both solvent and reductant. Precise control of surface energy between Pd and Pt precursors directs nucleation towards the icosahedral morphology, achieving a fourfold reduction in reaction time and an eightfold increase in yield relative to previous methods. The resulting nanocatalysts exhibit exceptional electrocatalytic activity, with a mass activity of 1.54 A mg^−1^ Pt at 0.9 V versus RHE—surpassing commercial Pt/C—and excellent durability after 10,000 potential cycles.

The icosahedral morphology, rich in {111} facets and twin boundaries, is known to maximize ORR turnover, while the Pd core reduces platinum loading without sacrificing activity. The one-pot route and high yield position this synthesis for industrial translation, making this contribution directly relevant to the commercialization of hydrogen fuel cells.

## 5. Bipolar Organic Electrode Nanomaterials for Lithium Batteries [31]

The replacement of transition-metal inorganic electrodes with organic molecules is a pivotal goal for sustainable, cobalt-free lithium batteries. Marinova et al. [31] investigate the electrochemical behavior of naphthalimide derivatives functionalized with a peri-dichalcogenide bridge as bipolar electrode materials in lithium half-cells with ionic liquid electrolytes. Combining cyclic voltammetry, impedance spectroscopy, and DFT calculations, the authors delineate the contribution of each molecular fragment: the carbonyl groups of the imide unit participate in multi-electron reduction below 2.0 V, while the chalcogenide bridge drives oxidation above 3.9 V via involvement of the electrolyte counter-ion TFSI^−^. This produces a true bipolar behavior—the same molecule acts as both a cathode and anode material.

The integration of DFT calculations with experimental electrochemistry provides a mechanistic foundation for molecular design of future bipolar organic electrodes. The results point towards a rich design space in peri-functionalized naphthalenimides, with potential for multi-electron storage and improved capacity density.

## 6. Graphene Oxide-Anchored Cu–Co Nanocatalysts for Electrochemical Nitrate Reduction [32]

Excess nitrate in water bodies—from agricultural runoff and industrial effluents—poses a critical threat to ecosystems and human health. Electrochemical nitrate reduction to ammonia offers a dual benefit: pollutant removal and renewable ammonia synthesis. Liu et al. [32] design graphene oxide-anchored bimetallic Cu–Co catalysts and demonstrate that the interface between the metallic and oxide phases in the Cu–Co heterostructure significantly enhances both activity and stability. The graphene oxide support promotes uniform nanoparticle dispersion, provides conductive pathways, and anchors the Cu–Co clusters through strong metal–support interactions. Characterization by electron microscopy and X-ray photoelectron spectroscopy confirms the formation of well-defined heterointerfaces, while electrochemical measurements show high Faradaic efficiency for ammonia production.

This work exemplifies the synergistic use of two-dimensional nanomaterials as catalytic supports and the rational design of bimetallic heterointerfaces, two themes central to the Section’s scope. The results contribute to the growing field of electrocatalytic nitrogen cycling with significant environmental implications.

## 7. Nanotoxicology: Silver Nanoparticles and Nanoplastics in a Human Neuronal Model [33]

As engineered nanomaterials and nanoplastics increasingly co-exist in the environment, understanding their combined effects on human cells has become an urgent priority. Brzóska et al. [33] investigate the co-exposure of undifferentiated and differentiated Lund human mesencephalic cells—a well-established neuronal model—to 20 nm silver nanoparticles and 20 nm polystyrene nanoparticles. While polystyrene nanoparticles alone had negligible effects on viability or gene expression, silver nanoparticles significantly reduced cell viability and dysregulated the expression of inflammation-related long non-coding RNAs. Strikingly, co-exposure to silver nanoparticles and high concentrations of polystyrene nanoparticles produced a synergistic reduction in viability in differentiated neurons and complex antagonistic and synergistic interactions at the long non-coding RNA expression level depending on differentiation status.

This article addresses a critical knowledge gap: most nanotoxicology studies examine single agents in isolation, yet real-world exposure inevitably involves mixtures. The cell-differentiation-dependent response highlights that neuronal development stage modulates nanoparticle toxicity—a finding with direct implications for risk assessment of nanotechnology in consumer products and environmental contamination.

## 8. Green Chemistry: Thiophene-Based Surfactants with Life Cycle Assessment [34]

The chemical industry’s transition to sustainable processes requires both green synthesis design and rigorous environmental impact quantification. Stoica et al. [34] synthesize three novel π-conjugated thiophene-based surfactants—an oligomer, a diblock copolymer, and a random copolymer—and evaluate their surfactant performance alongside full life cycle assessment. The life cycle assessment identifies synthetic hotspots—the reaction steps and solvents contributing most to energy use and ecotoxicity—and provides actionable guidance for redesigning the synthesis towards reduced environmental impact without compromising surface-active performance.

By embedding life cycle assessment directly into nanomaterial synthesis research, this work models a standard that the broader nanomaterials community would benefit from adopting. The thiophene-based surfactants themselves have potential in optoelectronic coatings and emulsification applications, while the methodology is immediately transferable to other functional nanomaterial synthesis routes.

## 9. Biopolymer–Graphene Nanocomposites for Biotechnology [35]

The combination of graphene’s exceptional electrical, mechanical, and optical properties with the biodegradability and biocompatibility of natural polymers is generating a versatile class of bio-nanocomposites with transformative potential in medicine and biotechnology. Binaymotlagh et al. [35] provide a comprehensive review of synthesis strategies and applications of graphene- and graphene oxide-based bio-nanocomposites, focusing on polysaccharide-based and protein-based matrices including chitosan, hyaluronic acid, cellulose, and silk fibroin. Applications surveyed span drug delivery, tissue engineering scaffolds, wound healing materials, antimicrobial systems, and industrial food applications. The review also addresses biodegradable synthetic polymers—PLA, PEG, and polyurethane—that bridge the gap between purely natural and conventional petroleum-based matrices.

The review’s systematic treatment of synthesis methods and structure–property relationships makes it an authoritative reference for researchers designing bio-nanocomposites. Its emphasis on sustainability—favoring biodegradable matrices and low-toxicity graphene oxide derivatives—positions it squarely within the evolving ethos of responsible nanotechnology.

## 10. Stabilizing Plasmonic Nanoparticles in Organic Solvents [36]

Localized surface plasmon resonance in metal nanoparticles underpins applications from biosensing and photothermal therapy to photocatalysis and solar energy harvesting, but citrate-synthesized gold–silver nanoshells aggregate immediately upon transfer from aqueous to organic media, severely limiting their processability. Magdon et al. [36] conduct a systematic study of ligand exchange strategies—using Triton X-100, sodium stearate, polyvinylpyrrolidone, and hydroxypropyl cellulose—to stabilize gold–silver nanoshells in ethylene glycol, tetrahydrofuran, dichloromethane, and toluene. They identify polymeric coating systems that stabilize the colloidal state through dipole–dipole and hydrogen-bonding interactions rather than electrostatic repulsion, preserving the plasmonic signature in each organic phase. Crucially, all identified ligands are non-toxic, expanding the range of biocompatible solvents accessible for LSPR-based applications.

This work fills an important methodological gap: many emerging applications of plasmonic nanoparticles—organic photovoltaics, polymer blends, and oil-phase sensing—require stability in non-aqueous environments. By mapping which ligand–solvent combinations succeed and why, the authors provide an actionable toolkit for the plasmonic nanoparticle community.

## 11. Carbon Nanochain/MWCNT Composites for Lithium–Oxygen Batteries [37]

Lithium–oxygen batteries theoretically offer gravimetric energy densities five to ten times that of lithium-ion technology; however, achieving both high capacity and stable cycling remains a formidable challenge. Womble et al. [37] report a simple yet elegant solution: incorporating mesoporous carbon nanochains into the macropores of multi-walled carbon nanotube cathodes. Multi-walled carbon nanotubes provide the conductive, high-surface-area backbone while their large macropores normally require substantial electrolyte fill, diluting gravimetric performance. Carbon nanochains, with an average mesopore diameter of approximately 100 nm, fill these macropores and create additional storage sites for the Li_2_O_2_ discharge product, substantially improving true volumetric and gravimetric capacities. The composite cathodes also exhibit enhanced cycle life, attributed to more uniform deposition and decomposition of Li_2_O_2_.

This work illustrates a general principle: hierarchical pore engineering using two complementary carbon nanomaterials can simultaneously address capacity, electrolyte management, and cycle stability in metal–air batteries. The simplicity of the mixing-and-casting fabrication route makes this approach readily scalable.

## 12. Freestanding TiO_2_ Nanotube Arrays for Dye-Sensitized Solar Cells [38]

Dye-sensitized solar cells offer a low-cost route to photovoltaic energy conversion, but their efficiency is constrained by electron transport and light-harvesting limitations in nanoparticle photoanodes. Han et al. [38] investigate the influence of structural configuration—specifically closed-ended versus open-ended freestanding TiO_2_ nanotube arrays—on power conversion efficiency. Open-ended arrays provide direct electron pathways and enhanced dye loading; their power conversion efficiency increases progressively when further decorated with carbon materials to improve electron transport and TiO_2_ nanoparticle scattering layers to increase light harvesting. The combination of open-ended nanotube morphology, carbon-enhanced conductivity, and light-scattering layers achieves a meaningful efficiency improvement over baseline configurations.

This systematic deconstruction of each performance-enhancing element—nanotube orientation, carbon additives, and scattering layer—provides a clear design hierarchy for optimizing dye-sensitized solar-cell photoanodes. The nanoarchitectural strategy has broader relevance to photocatalytic and photoelectrochemical systems where electron transport and light absorption must be simultaneously optimized.

## 13. Gadolinium Tungstate Nanoflakes for Multimodal Cancer Therapy [39]

Combining photodynamic therapy, radiotherapy, and imaging in a single injectable nanoplatform represents the forefront of precision oncology. Kang and Gerken [39] demonstrate that gadolinium tungstate (Gd_2_(WO_4_)_3_) nanoflakes functionalized with poly(glycerol) overcome the dispersibility limitations of dense Gd-based nanomaterials in aqueous media. The high atomic numbers of Gd and W enable X-ray-induced generation of reactive oxygen species, acting as a radiosensitizer. Loading the photosensitizer chlorin e6 onto poly(glycerol)-functionalized nanoflakes confers strong photocytotoxicity at chlorin e6 concentrations as low as 0.2 μg mL^−1^ under light irradiation, while the Gd content also provides MRI contrast.

This trimodal nanoplatform—photodynamic sensitizer, X-ray radiosensitizer, and MRI contrast agent—embedded in a biocompatible polymer shell exemplifies the power of multifunctional nanoarchitecture. The ability to attack tumors with two independent cytotoxic mechanisms while monitoring therapeutic response by imaging represents a clinically compelling theranostic concept.

## 14. Nanoparticles for Cultural Heritage Conservation [40]

Nanotechnology’s contributions extend beyond high-technology industries into the preservation of humanity’s documentary heritage. Wojech et al. [40] present a comparative study of paper strengthening using gelatin, Klucel G, and Tylose combined with deacidification using magnesium hydroxide nanoparticles. Experiments on Whatman model papers, both artificially acidified and iron-gall-ink-coated, evaluate breaking length, cellulose degree of polymerization, pH, optical properties, and SEM-EDX morphology before and after treatment. Tylose provides the greatest increase in breaking length; Klucel G imparts superior dimensional stability; and gelatin most significantly alters optical properties. Combining polymer strengthening with magnesium hydroxide nanoparticle deacidification in a single workflow reduces the number of interventions that fragile originals must undergo.

This article demonstrates that nanoparticle-assisted conservation is maturing into a practical, evidence-based practice. The systematic comparison of three polymer–nanoparticle combinations gives conservators the data they need to match treatment to the specific vulnerabilities of individual archival collections.

Reading these fourteen contributions together, several powerful overarching themes emerge. First, nanomaterial multifunctionality is pervasive. Whether a single a-IZTO film serving as both semiconductor channel and transparent electrode [27], a nanoflake platform that simultaneously photosensitizes, radiosensitizes, and provides MRI contrast [39], or a thiophene surfactant whose molecular architecture simultaneously drives surface activity and enables conjugated-polymer electronics [34], the defining goal is achieving multiple functions from a single, well-engineered nanoscale object.

Second, sustainability and societal responsibility are increasingly embedded into nanomaterial research from the outset. The life-cycle-assessment-integrated surfactant synthesis [34], the biodegradable polymer matrices for graphene nanocomposites [35], the nanoarchitectonic food-packaging framework aiming to reduce plastic waste [29], and the magnesium hydroxide deacidification nanoparticles protecting irreplaceable cultural heritage [40] all demonstrate that the field is moving beyond performance optimization alone towards full life cycle and social impact awareness.

Third, the biomedical application of nanomaterials is showcased at several levels of sophistication. Injectable nanobrachytherapy [28] and multimodal theranostic nanoflakes [39] represent the leading edge of cancer nanomedicine, while nanotoxicology [33] provides the risk-assessment data that responsible clinical translation requires. The drug-delivery potential of biopolymer–graphene composites [35] adds another dimension to the biomedical landscape.

Fourth, carbon nanomaterials in energy applications remain a defining pillar of the Section. The carbon nanochain/MWCNT composite addresses pore engineering in Li–O_2_ batteries (paper 11) [37], TiO_2_ nanotube arrays advance solar energy conversion (paper 12) [38], Pd@Pt icosahedra drive fuel cell ORR catalysis (paper 4) [30], and bipolar naphthalimide derivatives chart a path towards cobalt-free lithium batteries (paper 5) [31]. Together, these articles span the generation, storage, and conversion of clean energy at the nanoscale.

Finally, the Section’s methodological sophistication is striking. DFT calculations illuminate organic electrode reaction mechanisms [31]; life cycle assessment quantifies environmental footprints of synthetic routes [34]; advanced characterization techniques including XRD, XPS, SEM-EDX, and microscopy underpin mechanistic claims throughout; and the nanotoxicology work deploys transcriptomic endpoints that go far beyond classical viability assays [33]. This methodological depth is what elevates these articles from incremental contributions to genuinely influential works.

Locking to the future, health and wellbeing are key topics for the present and future in nanotechnology. The integration of nanotechnology into personalized medicine has reached a pivotal milestone, driven by convergent advances in nanocarrier engineering, artificial intelligence, and next-generation immunotherapy. Multifunctional nanoparticles that combine therapeutic and diagnostic roles—so-called theranostic agents—can deliver chemotherapeutics, imaging agents, and even gene-editing tools simultaneously, offering a powerful platform for precision oncology, where nanoparticle size, surface charge, shape, and functionalization are key determinants of biodistribution, tumor penetration, and systemic toxicity. A landmark clinical breakthrough came from LNP-mRNA neoantigen vaccine platforms: the randomized phase-2 KEYNOTE-942 trial reported a 2.5-year recurrence-free survival of 74.8% for mRNA-4157 plus pembrolizumab versus 55.6% for pembrolizumab alone in resected stage III/IV melanoma, while a *Nature* report on autogene cevumeran—an LNP-formatted poly-neoantigen vaccine for pancreatic ductal adenocarcinoma—showed that approximately 50% of recipients mounted durable CD8^+^ T-cell responses correlating with prolonged disease-free survival. Complementing these therapeutic advances, the integration of artificial intelligence and nanotechnology is reshaping cancer diagnosis and treatment through intelligent nanoplatforms that combine tumor sensing, targeted delivery, controlled release, and adaptive response within a single system, while AI analyzes large-scale multi-omics and clinical datasets to support early cancer detection and refinement of personalized treatment strategies. In terms of biological delivery, exosomes, as natural intercellular messengers, are gaining prominence as delivery vehicles in nanomedicine, offering a superior alternative to conventional synthetic nanoparticles by effectively encapsulating diverse therapeutic agents while exhibiting low toxicity, favorable pharmacokinetics, and organotropic properties. Biomimetic nanoplatforms that leverage natural cell membrane properties are also increasingly recognized for their potential to improve drug delivery and immune modulation, including TME-activated cancer cell membrane–liposome hybrid nanoparticles for synergistic metabolic and chemotherapy applications. Finally, the development of cancer-cell-targeted nanomedicines operating within the intricate tumor microenvironment represents a pivotal breakthrough, enabling precise interventions that address the critical challenges of drug delivery barriers across multiple cancer types in personalized oncomedicine. Together, these contributions represent the most exciting recent developments at the intersection of advanced nanomaterials and personalized medicine [41,42,43,44,45,46].

The fourteen Editor’s Choice articles highlighted here provide a vivid panorama of the “Advanced Nanomaterials and Nanotechnology” Section in 2025. They demonstrate that nanomaterials research is simultaneously deepening its mechanistic foundations, broadening its application domains, and maturing in its engagement with sustainability and safety. From transparent nanoelectronics and injectable nanobrachytherapy to cultural heritage conservation and sustainable food packaging, the Section spans the full arc from fundamental science to societal impact.

The Editorial Board congratulates the authors of these outstanding contributions and encourages the global nanomaterials community to read these open access articles in full at https://www.mdpi.com/journal/materials/editors_choice (accessed on 4 May 2026). We warmly invite high-quality submissions to the Section and look forward to featuring the next generation of Editor’s Choice articles that will continue to advance the frontiers of nanomaterials science.
